# Recognition of Antibiotic Resistance in Spontaneous Bacterial Peritonitis Caused by Escherichia coli in Liver Cirrhotic Patients in Civil Hospital Karachi

**DOI:** 10.7759/cureus.5284

**Published:** 2019-07-31

**Authors:** Pooja Deepak, Laila Tul Qadar, Rohan Kumar Ochani, Zahid Ali Memon, Syeda Anjala Tahir, Khalid Imran, Naresh Kumar Seetlani, Amanullah Abbasi, Mahaish Kumar, Piyar Ali

**Affiliations:** 1 Internal Medicine, Civil Hospital, Karachi, PAK; 2 Internal Medicine, Dow University of Health Sciences, Karachi, PAK; 3 Surgery, Civil Hospital Karachi, Dow University of Health Sciences, Karachi, PAK; 4 Internal Medicine, Civil Hospital Karachi, Dow University of Health Sciences, Karachi, PAK; 5 Internal Medicine: Gastroenterology, Civil Hospital Karachi, Dow University of Health Sciences, Karachi, PAK; 6 Internal Medicine: Infectious Disease, Civil Hospital Karachi, Dow University of Health Sciences, Karachi, PAK; 7 Internal Medicine, Aga Khan University Hospital, Karachi, PAK

**Keywords:** antibiotic-resistance, ascites, broad-spectrum antibiotics, end stage liver disease, escherichia coli (e. coli), paracentesis, spontaneous bacterial peritonitis

## Abstract

Introduction

Spontaneous bacterial peritonitis (SBP) is the most common life-threatening infection in patients with ascites due to liver cirrhosis. The infection is most commonly caused by the bacterium Escherichia coli, commonly referred to as E. coli. Over the past few years, the incidence of antimicrobial resistance against E. coli has risen drastically, leading to increased morbidity and mortality.

Methods

This cross-sectional study was conducted to determine the pattern of resistance using variations of antibiotics against E. coli, to prevent its empirical usage and initiate an appropriate target antibiotic therapy. The data were collected from May 2017 to October 2017 and included a total of 184 patients. The patients had previously been diagnosed with chronic liver disease and had presented with E. coli-induced SBP in the medicine wards at Civil Hospital, Karachi, which is the largest tertiary care hospital in the city. All participants underwent diagnostic paracentesis, and the ascitic fluid samples were sent to labs for culture and sensitivity to antibiotics.

Results

The sample population consisted of 184 participants, of which two-thirds (63.6%; n=117/184) of the population consisted of males. The mean age of the participants was 47.6±10.7 years. More than half of the patients had hepatitis C (54.9%; n=101/184) while the remaining were diagnosed with hepatitis B (45.1%; n=83/184). The ascitic fluid showed varying percentages of resistance for drugs, with no resistance to imipenem and meropenem while ciprofloxacin showed the highest resistance in eradicating the bacterium, E. coli. Additionally, a statistical correlation was tested between drug resistance and factors like age, gender, duration of liver disease, and duration of ascites. Ciprofloxacin and tetracycline showed a positive correlation between the resistance of these drugs and the age, gender, and duration of chronic liver disease in the participants while trimethoprim/sulfamethoxazole, amoxicillin/clavulanic acid, and piperacillin/tazobactam showed a positive association with the duration of ascites.

Conclusion

A rapid diligent intervention of cirrhotic patients with complicated ascites is crucial to alleviate patient mortality. Due to the rising bacterial resistance, primarily, epidemiological patterns should be assessed and analyzed in our regional hospitals, and then, antibiotics should be prescribed meticulously.

## Introduction

Ascites is the most common complication in patients with liver cirrhosis or end-stage liver disease (ESLD), consisting of more than 50% of the cases [1]. Cirrhotic patients, due to ascites, develop spontaneous bacterial peritonitis (SBP), which is the most common life-threatening infection of ascitic fluid. Its incidence ranges from 10% to 30% [2] and has several variable etiologies, with the spreading of bacteria from the gut to the lymph nodes being the most common underlying pathogenesis [3].

Escherichia coli (E. coli), an intestinal gram-negative bacterium, is known to be a significant cause of SBP, with non-enterococcus streptococci being the second [4]. According to a study conducted in 2011, the frequency of E. coli isolated from ascitic fluid in SBP was 66.6%, which went up by three times from 22% in 2010 [5-6].

SBP has a varying clinical presentation, ranging from being asymptomatic to developing symptoms like abdominal pain, fever, nausea, and diarrhea. The disease can also lead to deteriorating symptoms, such as increased confusion and encephalopathy [7]. The diagnosis of SBP is classically made by looking at the ascitic fluid’s neutrophil count of more than or equal to 250 cells/mm^3^ in the presence of a positive culture [8]. However, the culture usually takes four to five days to confirm the results, leading to a rise in empirical treatment and delayed targeted therapy. This has caused the mortality rate of SBP to rise drastically despite its early diagnosis and prompt management [9].

SBP has a staggering mortality rate of 40%-70% in cirrhotic patients and further rises to 80% for patients who develop septic shock secondary to the infection [10]. Additionally, the constant neglect in starting definitive therapy has led the mortality rate to rise from 10% to 46% [11-12]. Therefore, there is a crucial need to recognize the antimicrobial resistance pattern of E. coli so that the targeted antibiotic therapy can be started more readily. Hence, we conducted this study to determine the frequency of patterns of antibiotic resistance in cases of SBP caused by E. coli in patients with liver cirrhosis presented in a tertiary care hospital of Karachi, Pakistan.

The practice of empirical treatment has increased the antimicrobial resistance to certain drugs, making the eradication of the organisms causing SBP a major challenge. A review by Sola et al. showed the rising failure of conventional treatment options, such as third-generation cephalosporin (TGC) [13], although local studies have shown no such problems [14].

## Materials and methods

This cross-sectional study was carried out in Karachi, Pakistan, from May 2017 to October 2017. The sample population consisted of male and female patients aged 12 years and above. The patients presented to Civil Hospital Karachi and only patients with previously diagnosed liver cirrhosis were chosen, irrespective of their precipitating etiology. They had a Child-Pugh score of B or C and currently presented with the complaint of painful abdominal distention with or without fever. A non-probability consecutive technique was used to calculate the sample size, keeping a confidence level of 95% and a margin of error of 7%.

The study only included subjects who presented with symptoms of SBP, which are painful abdominal distention in the presence or absence of fever and were diagnosed with abdominal ascites on ultrasound (U/S) abdomen at the time of presentation. Patients only diagnosed with community-acquired SBP were included. Informed verbal consent was also taken from all participants before enrolling them in the study.

The diagnosis of chronic liver disease (CLD) was made based on clinical examination and radiological evidence. Participants with a precipitating etiology other than CLD, such as nephrotic syndrome and congestive heart failure, were excluded. Moreover, patients who were diagnosed with SBP and were either on antibiotics or had received antibiotics in the past 10 days, as well as patients who had a poly-microbial growth in the ascitic fluid culture, were also excluded.

According to the symptoms and the patient’s significant history of liver disease, a high index of suspicion for ascites complicated with SBP was kept in mind. To confirm the diagnosis of ascites, patients underwent a departmental U/S, and then they were sent for paracentesis. The procedure was performed by a qualified postgraduate trainee doctor who followed standard precautionary guidelines and used all the aseptic techniques.

The procedure was as follows: a total of 20 ml ascitic fluid was collected from each patient for the detailed report (DR) and culture and sensitivity (CS). Ten ml of ascitic fluid was placed in blood culture bottles at the bedside using an aseptic technique. Three ml of the sample was directly cultured on two separate blood agar plates and a chocolate agar plate, after centrifuging and incubating it at 37°C. The culture plates were monitored daily for the next two days to identify any significant growth by a qualified microbiologist of a tertiary care hospital laboratory. Resistance was monitored against various antibiotics: ampicillin/sulbactam, trimethoprim/ sulfamethoxazole (co-trimoxazole), piperacillin, ciprofloxacin, tetracycline (doxycycline), amikacin, ofloxacin, ampicillin, amoxicillin/ clavulanic acid, ceftriaxone, piperacillin/tazobactam, imipenem, and meropenem.

The final results of the ascitic routine examination were made available within a day while the culture and sensitivity report was made available after one week. Until then, the patients were treated empirically with broad-spectrum antibiotics, and the response of the therapy was evaluated using the patients’ symptoms.

Data were entered and analyzed using IBM SPSS Statistics v. 22.0 (Armonk, NY, US). Descriptive statistics were used to report frequencies and proportions for the categorical responses. Variables such as gender, age, duration of CLD, and duration of ascites were stratified to see the effect of these variables on E. coli-mediated resistance against the aforementioned drugs. T-test was applied for post-stratification, and a p-value of ≤ 0.05 was taken as significant.

## Results

**Figure 1:**
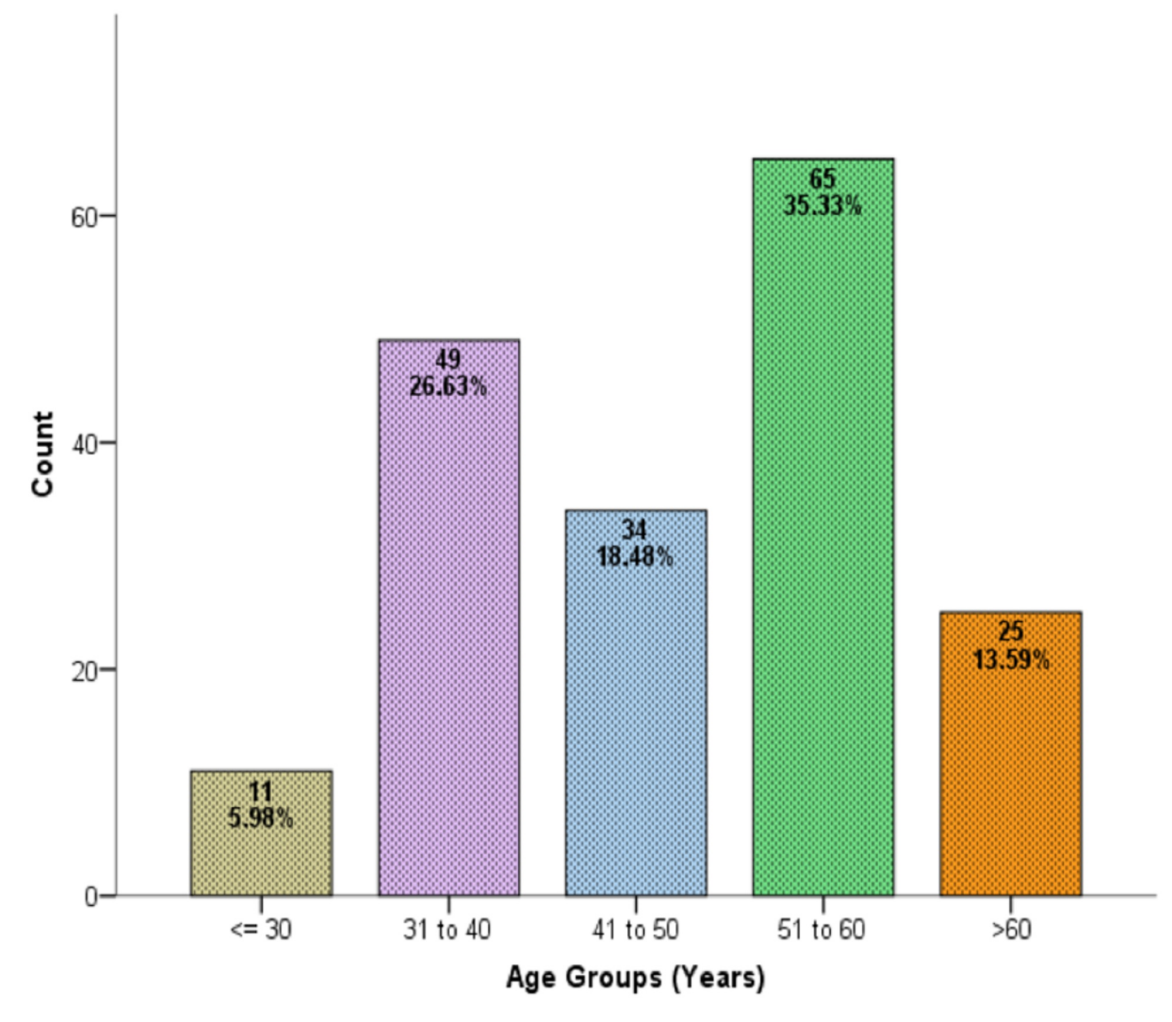
Age distribution of the patients

The sample population consisted of 184 patients, of which 117 (63.6%) were males and 67 (36.4%) were females, with a mean age of 47.6 ± 10.7 years. Of the 184 participants, about half of them were diagnosed with hepatitis B (45.1%; n=83/184) and more than half had hepatitis C (54.9%; n=101/184). Figure 1 shows the age distribution of the patients.

A confirmed diagnosis was made utilizing the ascitic fluid detailed report while the organism was identified by performing ascitic fluid culture and sensitivity. The most common organism on culture was E. coli; hence, the drugs used to treat SBP due to E. coli had various resistance patterns, which is shown in Table 1. Amongst all the drugs, the highest resistance was seen with ciprofloxacin and ampicillin, accounting for 38.6% and 37.5%, respectively, while no resistance was seen with the use of imipenem and meropenem.

**Table 1:** Frequency of antibiotic resistance in spontaneous bacterial peritonitis caused by E. coli in patients with liver cirrhosis

ANTIBIOTICS	RESISTANCE	
	N/184	%
Ampicillin/Sulbactam	5	2.7%
Trimethoprim/Sulfamethoxazole	20	10.9%
Piperacillin	1	0.5%
Ciprofloxacin	71	38.6%
Tetracycline	52	28.3%
Amikacin	6	3.3%
Ofloxacin	24	13%
Ampicillin	69	37.5%
Amoxicillin/Clavulanic acid	33	17.9%
Ceftriaxone	59	32.1%
Cefotaxime	22	12%
Piperacillin/Tazobactam	12	6.5%
Imipenem	0	0%
Meropenem	0	0%

Table 2 shows the frequency of antibiotic resistance in SBP caused by E. coli and its association with age groups. Ciprofloxacin, tetracycline, and ampicillin expressed the highest resistance in participants less than 50 years of age. Of which, ciprofloxacin (p=0.023) and tetracycline (p=0.035) showed a significant correlation between the resistance of these certain drugs and age groups.

**Table 2:** Frequency of antibiotic resistance in spontaneous bacterial peritonitis caused by E. coli in patients with liver cirrhosis by age groups

ANTIBIOTICS	RESISTANCE		P-Value
	Age ≤50 Years (N = 94)	Age >50 Years (N = 90)	
	N (%)	N (%)	
Ampicillin/Sulbactam	4(4.3%)	1(1.1%)	0.190
Trimethoprim/Sulfamethoxazole	7(7.4%)	13(14.4%)	0.127
Piperacillin	1(1.1%)	0(0%)	0.085
Ciprofloxacin	44(46.8%)	27(30%)	0.023
Tetracycline	33(35.1%)	19(21.1%)	0.035
Amikacin	3(3.2%)	3(3.3%)	0.99
Ofloxacin	11(11.7%)	13(14.4%)	0.581
Ampicillin	33(35.1%)	36(40%)	0.493
Amoxicillin/Clavulanic acid	12(12.8%)	21(23.3%)	0.062
Ceftriaxone	28(29.8%)	31(34.4%)	0.499
Cefotaxime	10(10.6%)	12(13.3%)	0.573
Piperacillin/Tazobactam	4(4.3%)	8(8.9%)	0.203
Imipenem	0(0%)	0(0%)	NA
Meropenem	0(0%)	0(0%)	NA

Table 3 shows the frequency of antibiotic resistance in SBP caused by E. coli and its association with gender. The resistance of ciprofloxacin and ampicillin were highest in the male gender while females had the highest resistance to ampicillin and ceftriaxone. Ciprofloxacin (p=0.007), tetracycline (p=0.002), and piperacillin/tazobactam (p=0.007) showed a significant correlation between antibiotic resistance and gender.

**Table 3:** Frequency of antibiotic resistance in spontaneous bacterial peritonitis caused by E. coli in patients with liver cirrhosis by gender

ANTIBIOTICS	RESISTANCE		P-Value
	Male n=117	Female n=67	
	n (%)	n (%)	
Ampicillin/Sulbactam	3(2.6%)	2(3%)	0.866
Trimethoprim/Sulfamethoxazole	13(11.1%)	7(10.4%)	0.889
Piperacillin	0(0%)	1(1.5%)	0.364
Ciprofloxacin	54(46.2%)	17(25.4%)	0.007
Tetracycline	28(41.8%)	24(20.5%)	0.002
Amikacin	6(5.1%)	0(0%)	0.059
Ofloxacin	18(15.4%)	6(9%)	0.213
Ampicillin	42(35.9%)	27(40.3%)	0.533
Amoxicillin/Clavulanic acid	22(18.8%)	11(16.4%)	0.685
Ceftriaxone	38(32.5%)	21(31.3%)	0.874
Cefotaxime	17(14.5%)	5(7.5%)	0.155
Piperacillin/Tazobactam	12(10.3%)	0(0%)	0.007
Imipenem	0(0%)	0(0%)	NA
Meropenem	0(0%)	0(0%)	NA

Table 4 shows the frequency of antibiotic resistance in SBP caused by E. coli and its association with the duration of CLD. Several antibiotics had a positive association with the duration of CLD, where ciprofloxacin and ceftriaxone had the highest resistance in participants with a duration of less than four years.

**Table 4:** Frequency of antibiotic resistance in spontaneous bacterial peritonitis caused by E. coli in patients with liver cirrhosis by duration of chronic liver disease

ANTIBIOTICS	RESISTANCE		P-Value
	Duration of chronic liver disease ≤ 4 Years n= 128	Duration of chronic liver disease > 4 Years n= 56	
	n (%)	n (%)	
Ampicillin/Sulbactam	4(3.1%)	1(1.8%)	0.607
Trimethoprim/Sulfamethoxazole	18(14.1%)	2(3.6%)	0.035
Piperacillin	1(0.8%)	0(0%)	0.507
Ciprofloxacin	59(46.1%)	12(21.4%)	0.002
Tetracycline	27(21.1%)	25(44.6%)	0.001
Amikacin	5(3.9%)	1(1.8%)	0.456
Ofloxacin	6(4.7%)	18(32.1%)	0.0005
Ampicillin	39(30.5%)	30(53.6%)	0.003
Amoxicillin/Clavulanic acid	31(24.2%)	2(3.6%)	0.001
Ceftriaxone	47(36.7%)	12(21.4%)	0.041
Cefotaxime	10(7.8%)	12(21.4%)	0.009
Piperacillin/Tazobactam	7(5.5%)	5(8.9%)	0.382
Imipenem	0	0	NA
Meropenem	0	0	NA

Table 5 shows the frequency of antibiotic resistance in SBP caused by E. coli and its relation to the duration of ascites. A high resistant pattern was seen by ciprofloxacin, tetracycline, ceftriaxone, and ampicillin in both the groups, although a significant correlation was found between the drugs trimethoprim/sulfamethoxazole (TMP-SMX), amoxicillin/clavulanic acid, and piperacillin/tazobactam and the duration of ascites.

**Table 5:** Frequency of antibiotic resistance in spontaneous bacterial peritonitis caused by E. coli in patients with liver cirrhosis by duration of ascites

ANTIBIOTICS	RESISTANCE		P-Value
	Duration of Ascites ≤ 24 h n= 162	Duration of Ascites > 24 h n= 22	
	n (%)	n (%)	
Ampicillin/Sulbactam	3(1.9%)	2(9.1%)	0.109
Trimethoprim/Sulfamethoxazole	13(8%)	7(31.8%)	0.004
Piperacillin	1(0.6%)	0(0%)	0.988
Ciprofloxacin	61(37.7%)	10(45.5%)	0.481
Tetracycline	42(25.9%)	10(45.5%)	0.056
Amikacin	4(2.5%)	2(9.1%)	0.101
Ofloxacin	24(14.8%)	0(0%)	0.053
Ampicillin	62(38.3%)	7(31.8%)	0.557
Amoxicillin/Clavulanic acid	25(15.4%)	8(36.4%)	0.016
Ceftriaxone	49(30.2%)	10(45.5%)	0.152
Cefotaxime	17(10.5%)	5(22.7%)	0.097
Piperacillin/Tazobactam	5(3.1%)	7(31.8%)	0.005
Imipenem	0	0	NA
Meropenem	0	0	NA

## Discussion

ESLD carries a global burden for health care, with an estimated 1.2 million mortalities [15-16]. Patients with ESLD pose a weaker immune system and, therefore, have increased exposure to bacterial attacks, sepsis, and death. Bacterial infections in patients with liver cirrhosis are not only more frequent but also are responsible for the patient’s deteriorating condition.

SBP, with a prevalence of 8%-27% in patients with cirrhotic ascites, remains amongst the most significant complication in these patients [17]. Most SBP cases (70%) are caused by gram-negative bacteria, most commonly due to E. coli. As seen similarly to our results, using the mechanism of enterobacteria trans-locating from the intestinal tract [18].

Past literature has shown a four-fold increase in the mortality of patients reaching almost 58% in one month and 63% in 12 months [19]. One of the significant reasons for such high mortality is the rising antimicrobial resistance to antibiotics, including cephalosporin; especially, cefotaxime, fluoroquinolones, ampicillin/sulbactam, and aminoglycosides [20]. The dire consequences require prompt diagnosis and appropriate treatment of the infection when managing patients with liver cirrhosis [21].

Moreover, our study showed that 55% of the participants had hepatitis C as the identifiable cause of their liver disease. This was in accordance with a study done by Toshikuni N et al., where 67%-91% of the participants were diagnosed with hepatitis C as the primary etiology of cirrhosis [22].

Furthermore, our results showed a significantly elevated (38.6%) resistance to ciprofloxacin against the eradication of E. coli. This is in congruence with a study conducted by Ortiz et al. where E. coli showed resistant to the use of fluoroquinolones and their failure as a successful prophylactic agent against SBP [23]. Similarly, another study done in Spain enumerated an increased probability in the occurrence of SBP in patients who took fluoroquinolones as a prophylactic agent, concluding intravenous ceftriaxone as a more efficacious drug for SBP prophylaxis [24]. The reasons demonstrated behind the failure of oral fluoroquinolones were primarily due to its slow onset and changing the epidemiology of infections [24]. Although, contrary to our results, a study done in 2017 showed that the intake of oral fluoroquinolones was successful in preventing and treating SBP [25].

Conversely, studies conducted in Italy and France manifested reduced efficacy with the use of TGC for SBP with a failure rate of 41% and 57%, respectively [26-27]. However, our study showed a bacterial resistance of 32.1%, which was far less than the proportion of resistance in the west.

In the present study, sensitivity to piperacillin-tazobactam, amikacin, ampicillin/sulbactam, imipenem, and meropenem was better with imipenem and meropenem being 0%, showed by Lutz P et al. as well [28]. Hence, several studies have recommended their usage as initial treatment as well as in the prophylaxis of SBP caused by gram-negative organisms [28-29].

In the year 2000, only 1.2% of bacteria were resistant to TGCs. Today, unfortunately, due to the rising antibiotic resistance, several studies have shown a rise in multidrug-resistant (MDR) bacterial infections in cirrhotic patients. A study conducted in 2012 discovered a swift rise from 10% to 23% in the prevalence of MDR bacterial infections between 1998 and 2011 [17], therefore, making it crucial for all medical professionals to meticulously choose antibiotics and ultimately reducing the aftermaths of the increasing resistant pattern.

Having said that, our study had some limitations that need to be considered. Since only patients from a single tertiary-care hospital were specifically recruited in the study, the results of this study cannot be extrapolated to other hospitals. Therefore, further studies should be conducted in order to determine the generalizability of the results. Additionally, we excluded patients who were diagnosed with nosocomial SBP. Nevertheless, the findings of our study are vital in assessing the role of antibiotic resistance in the treatment of ESLD-induced SBP and the need for targeted therapy in the Pakistani population.

## Conclusions

Early and effective antimicrobial treatment is crucial in the management of cirrhotic patients with superimposed bacterial ascites. The choice of an empirical therapy should be based not only on the severity and origin of the infection but also on the local microbiological profile. In the absence of microbiological investigations that demonstrate the presence of gram-negative MDR bacteria, the carbapenems should not be empirically administered unless there is an established risk of MDR bacteria. The administration of inappropriate therapy is associated with increased mortality; hence, the epidemiological patterns of bacterial resistance in our regional hospitals should be evaluated and monitored.
